# TruMPET: A New Method for Protein Secondary Structure Prediction Using Neural Networks Trained on Multiple Pre-Selected Physicochemical and Structural Features

**DOI:** 10.3390/ijms262311284

**Published:** 2025-11-21

**Authors:** Yury V. Milchevskiy, Galina I. Kravatskaya, Yury V. Kravatsky

**Affiliations:** Engelhardt Institute of Molecular Biology, Russian Academy of Sciences, Vavilov Str., 32, 119991 Moscow, Russia; gk@eimb.ru (G.I.K.); jiri@eimb.ru (Y.V.K.)

**Keywords:** protein secondary structure, PSSP (protein secondary structure prediction), DSSP (Dictionary of Secondary Structure in Proteins), machine learning (ML), LDA (Linear Discriminant Analysis), ncAA (non-canonical amino acid)

## Abstract

Protein structure prediction continues to pose multiple challenges, despite the progress made by ML. While recent deep learning models have achieved a strong performance using embeddings from protein language models, they often ignore non-canonical amino acids and rely heavily on sequence alignments or evolutionary profiles. Here, we present an improvement to this approach for predicting the secondary protein structure of DSSP classes solely from amino acid sequences. We suggest that ML feature sets should be generated from statistically significant mutually uncorrelated descriptors. The selection of statistically assessed descriptors, including predicting the physicochemical parameters of non-canonical amino acids, is a key component of the proposed method. The statistical significance and influence of each of the suggested features were assessed using a two-step Linear Discriminant Analysis, which permitted the evaluation of the statistical significance of each descriptor and their impact on model accuracy. We applied the set of 109 most influential statistically significant descriptors as a learning model for the two-layer Bi-LSTM network combined with ESMFold2 embeddings. Our method, TruMPET (Training upon Multiple Pre-selected Elements Technique), outperformed all other methods reported in the literature for the non-redundant datasets (CB513: DSSP Q3 = 91.36% and Q8 = 85.41%, TEST2018: DSSP Q3 = 90.64% and Q8 = 84.17%).

## 1. Introduction

Proteins are biological macromolecules that constitute approximately 57% of a cell’s dry mass [1] and serve as the fundamental structural basis of life. Determining the precise structure of a protein typically involves crystallization followed by complex experimental procedures [2]. However, crystallization is not always feasible [3], and even when crystallization is successful, resolving the structure of the crystallized protein remains a non-trivial and often ambiguous process—particularly for large proteins [4]. Consequently, the prediction of a protein’s structure from its amino acid sequence has long been, and continues to be, one of the central challenges in modern biological science.

Given that this has been a central challenge in biophysics and structural biology for over six decades, and that it remains an area of active research, it is not feasible to provide a comprehensive review of all existing methods within the scope of this work. Interested readers are instead referred to reviews such as [5,6], which offer structured overviews of the methodologies that have been historically employed and continue to be developed for protein structure prediction. A detailed summary of the current state of the field can be found in [7].

At present, it may appear that protein secondary structure prediction (PSSP) has lost its relevance considering recent advances in tertiary structure prediction achieved by state-of-the-art machine learning approaches such as those in [8,9,10]. Nevertheless, accurate PSSP remains a cornerstone challenge in structural bioinformatics, with wide-ranging implications for protein function annotation, fold recognition, and structure-based design:

- PSSP continues to be the foundation for understanding the tertiary structure of proteins [11].
- PSSP and the subsequent study of the protein’s predicted secondary structure can improve the accuracy of tertiary structure predictions [12,13,14,15].
- Employing PSSP can divide amino acid sequence data into clearly recognizable patterns—helices, strands, and coils—which often correlate with functional domains [16,17].
- PSSP remains important in cases when structural confidence is lower or for interpreting dynamic and disordered regions erroneously captured by tertiary structure prediction methods [18].
- PSSP continues to play a crucial role in resolving protein functions and properties, as this structure is the basis for the formation of the tertiary structure [19].
- PSSP can be a low-cost and efficient alternative to wet experiments, making it particularly valuable for large-scale proteomic studies and drug discovery applications where experimental protein structure recovery can be prohibitively expensive and time-consuming [11]. For the same applications, if homologous structural data are unavailable, PSSP modeling is the only way to enable large-scale screening in silico [20].

Recent advances in protein language models (e.g., ESM2 [8], ProtTrans [21]) and deep learning architectures have led to state-of-the-art results in secondary structure prediction, often bypassing manual feature engineering through the direct processing of amino acid sequences [22].

A possible approach to PSSP is to first predict the protein’s tertiary structure using AlphaFold [9] and subsequently derive its secondary structure with tools such as mkdssp [23]. It should be noted, however, that although AlphaFold demonstrates remarkable overall performance, it exhibits reduced accuracy for certain CASP14 targets, particularly in flexible protein regions and subunits of multiprotein complexes. According to the authors of the method, high prediction confidence (pLDDT > 90) was achieved in only 35.7% of cases, while confident predictions (pLDDT > 70) accounted for 58.7% of cases [24]. The limitations of AlphaFold2 and potential strategies to address them are discussed in detail in [25,26].

The considerations outlined above underscore the potential value of methods capable of predicting protein structures without relying on multiple sequence alignments, particularly for synthetic proteins or those lacking homologs with experimentally determined structures. Consequently, the development of diverse approaches for PSSP remains an active and relevant area of research, even in the post-AlphaFold era [27,28,29,30,31,32,33,34,35,36,37,38,39,40,41,42].

Unfortunately, the substantial majority of protein structure prediction methods do not include special treatment of non-canonical amino acids, which are either mapped to canonical amino acids or discarded altogether. However, incorporation of non-canonical amino acids in proteins is not so rare—the proteins in the Protein Data Bank [43] include more than 1000 distinct non-canonical amino acids. If a learning model relies on the physicochemical properties of amino acids and if the amount of non-canonical amino acid residues in the certain protein is substantial (such as in hydroxyproline, which is more prevalent than proline in collagen-like protein chains [44] and stabilizes their structure [45]), then the structural prediction will inevitably be incorrect as these properties differ significantly from those of the canonical amino acid. To address this limitation, we integrated physicochemical properties from AAindexNC [46], a recent framework for estimating the physicochemical properties of non-canonical amino acids. This approach preserves the structural discrepancies between canonical and non-canonical amino acids that are otherwise lost.

Although recent advances in deep learning and protein language models (e.g., ESM2 [8], ProtTrans [21]) have minimized the reliance on manual feature engineering, physicochemical properties continue to be an important way to improve prediction accuracy and biological interpretability. Several studies have demonstrated that incorporating additional features based on physicochemical and/or structural properties such as hydrophobicity, charge, polarity, or flexibility can enhance neural network results [37,47]—particularly in challenging cases involving low-homology regions or structurally ambiguous fragments [48,49]. More recent works have confirmed that hybrid models combining pre-computed embeddings with biophysically grounded features can outperform purely sequence-based approaches, especially in downstream structural tasks [50,51,52,53]. Such descriptors can encode biochemical constraints that are not always captured by self-supervised training alone, and thus could be a complementary source of inductive bias for secondary and tertiary structure predictions [54].

The objective of this study was to generate a feature set that integrates all well-founded approaches to descriptor system design, as previously detailed in [55]. This involved the development of a set of statistically significant, mutually uncorrelated descriptors derived from the physicochemical and structural properties of amino acids, including non-canonical ones. To achieve the highest possible accuracy in secondary structure prediction, we utilized pre-computed ESM2 embeddings, followed by fine-tuning using a two-layer bidirectional LSTM neural network. Notably, our method does not rely on sequence alignment or homologous templates. In contrast to traditional profile-based pipelines, we employ a fully alignment-free strategy which implies that predictions are generated solely from structural element statistics, intrinsic physicochemical features, and contextualized amino acid embeddings.

## 2. Results

In this study, we achieved the highest accuracy in protein secondary structure prediction for alignment-free methods in comparison with the literature. We obtained this result in a manner that is interpretable and scientifically explainable, contributing to a deeper understanding of diverse protein structures. This was accomplished through the development of an objective, scientifically grounded system including stringent pre-selection and subsequent statistical analysis of descriptors that constitute the machine learning model.

### 2.1. Descriptor Pre-Selection Can Substantially Improve Prediction Quality

Predictors were generated in two stages: In the first stage, statistically significant and mutually non-correlated predictors were identified using Stepwise Discriminant Analysis [56,57], followed by Linear Discriminant Analysis (LDA) [58,59]. The stepwise approach to predictor selection is described in detail in [55], and its application in the context of protein structure prediction using neural networks is presented in [47].

**Figure 1:**
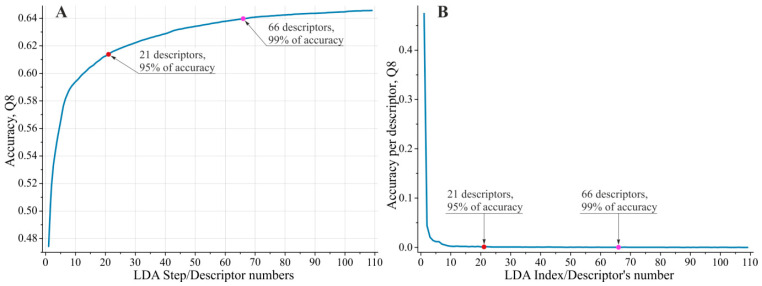
(**A**) Improvement in PSSP accuracy as a function of the number of descriptors selected by LDA; (**B**) individual contribution of each descriptor to the accuracy achieved by the complete feature set. In both graphs, the red and purple dots mark the top 21 and 66 descriptors, contributing ~95% and ~99% of the model’s predictive accuracy, respectively.

Descriptor pre-selection yielded biologically meaningful results, with the top 109 descriptors (listed in Supplementary Table S1) being readily interpretable. The top 109 descriptors selected by the ‘greedy’ Linear Discriminant Analysis (LDA) [60] procedure provided a substantially more precise model compared to the Stepwise Discriminant Analysis (SDA) [56,57] procedure. For the eight DSSP [61] classes, the Q8 accuracy reached 64.6%, compared to 50.5% for the best SDA model tested. This performance gap clearly justified the application of the two-stage descriptor pre-selection strategy. We selected the top 109 LDA-substantiated descriptors, as they capture virtually all (99.99%) of the predictive capacity of the feature set when applied within the ‘greedy’ selection algorithm (the impact of including the 110th descriptor on the resulting model accuracy was less than 0.005%). Figure 1 illustrates the improvement in PSSP accuracy as a function of the number of descriptors selected by LDA, as well as the individual contribution of each descriptor to the accuracy achieved by the complete feature set.

Analysis of the descriptors retained by LDA for subsequent modeling revealed that they cluster into three groups, with the top 21 descriptors contributing approximately 95% of the model’s predictive accuracy and the top 66 descriptors providing 99% (see Supplementary Table S1). These groups can be characterized as follows:

- RMSD-based structural descriptors;
- Physicochemical-based descriptors, describing specific periodicities of the protein secondary structure;
- Physicochemical- and structure-based descriptors, capturing non-periodic properties that influence the formation of the protein backbone configuration.

#### 2.1.1. RMSD-Based Structural Descriptors

To generate descriptors based on the statistics of conformational occurrence, we employed protein blocks (PBs) as defined by de Brevern [62,63,64]. We assessed the distribution of RMSD distances between fragments with identical sequences to each PB. From these distributions, it is possible to generate probability-based descriptors through nonlinear transformations, e.g., for the pentapeptide “PPPPP”, the RMSD distances to PB “m” (which exhibits a conformation close to the idealized *α*-helix) for all fragments from the training dataset are significantly higher than the average across the training dataset with relatively low variance. Based on this result, a straightforward descriptor can be proposed for α-helix classification: the pentapeptide “PPPPP” is definitely not an alpha helix.

However, even in this case, a problem arises with the quantity of identical sequences for statistical analysis. It is evident that for a pentapeptide (an amino acid sequence with a length of five residues), there are 20^5^ = 3.2 × 10^6^ possible combinations. Consequently, some sequence combinations are significantly underrepresented in the training dataset. This sparsity prevents us from acquiring reliable structural statistics for many sequence patterns.

To address this issue, we developed so-called reduced alphabets, in which certain amino acids are treated as equivalent. Using this definition, sequence fragments can be described as regular expressions, e.g., amino acids belonging to classes such as aliphatic amino acids (GAVLI), sulfur-containing amino acids (CM), aromatic amino acids (YWF), and charged amino acids can be considered identical within their class. These equivalence rules may vary depending on the position of the residue within the fragment, as central residues, for instance, often exert a stronger impact on the predicted structure. We systematically evaluated a wide range of reduced alphabets according to their influence on prediction performance, ultimately selecting a limited set. These reduced alphabets also differ in terms of the length of the fragment considered. In some cases, we successfully applied reduced alphabets for fragment lengths as large as 11 residues. All reduced alphabets employed in this study are provided in Supplement S2. References [65,66] are cited in the Supplementary Materials.

Let us consider how a descriptor based on a reduced alphabet is generated, e.g., a descriptor derived from t-statistics (i.e., based on the comparison of two sample means). In this approach, one must evaluate the probability that the mean value of one sample exceeds that of another while taking into account the respective sample sizes. For this case, the first sample consists solely of fragments of a given sequence *seq*, whereas the second sample comprises all remaining fragments.

To illustrate this point, let us consider one of the sixteen protein blocks PB*j*, *j* = [1…16] and *seq*, which is a given sequence of length 5. Let us introduce the following notation:

- *N*_occ_(*seq*)—the number of occurrences of *seq* among the sequences with known structures (i.e., the training dataset);
- $\bar{\mu_{j}}(seq)$—the mean distance between the structures with sequence *seq* and the PB*_j_*;
- $\bar{\mu_{j}}$—the average distance between PB*_j_* and all pentapeptides in the training dataset;
- $\sigma_{j}^{2}$—the sampling variance of PB*_j_*;
- $\sigma_{j}^{2}(seq)$—the variance for structures with sequence *seq*;
- *N*—the size of the training dataset.

Then, the t-statistics-based descriptor can be written as

(1) $$t_{j}(seq)=\frac{\bar{\mu_{j}}-\bar{\mu_{j}}(seq)}{\sqrt{s_{j}(seq)}},$$

where

(2) $$s_{j}(seq)=\frac{\sigma_{j}^{2}(seq)}{N_{\mathrm{occ}}(seq)}+\frac{\sigma_{j}^{2}}{N}$$

Note that when *N* ≫ *N*_occ_(*seq*) > 1,

(3) $$s_{j}(seq)≅\frac{\sigma_{j}^{2}(seq)}{N_{\mathrm{occ}}(seq)}$$

so (1) folds to

(4) $$t_{j}(seq)≅\frac{\bar{\mu_{j}}-\bar{\mu_{j}}(seq)}{\sigma_{j}(seq)}\sqrt{N_{\mathrm{occ}}(seq)}$$

Based on the value of the t-criterion *t_j_*(*seq*), we can assess the probability that for the pentapeptide *seq* protein block PB*_j_* is closer than the sample average.

In addition to this type of descriptor, we also used more complex descriptors that assessed the probability that a given amino acid sequence folds into the specific PB sequence, e.g., with the notation applied to describe PB [a, b,…,o], we successfully designed a descriptor that estimated the probability of occurrence of PB sequences such as ‘ddfmm’ or ‘ddddddd’ for a certain reduced alphabet. A complete description of all RMSD-based descriptors is provided in Supplement S2. The input data for this and all RMSD-based descriptors are also the obtained values for $\bar{\mu_{j}}(seq),$ $\bar{\mu_{j}},\;$$\sigma_{j}^{2}(seq),\;$$\sigma_{j}^{2}$ and *N*_occ_(*seq*).

Table 1 presents the most statistically significant RMSD-based descriptors.

#### 2.1.2. Descriptors, Describing Periodicities of PSSs

Among the descriptors capturing the periodicity in amino acid properties, we identified periods close to the canonical values of 3.6 and 3.0, which are consistent with the well-established periodic patterns of α-helices and 3_10_-helices. As described in Section 4.5 Descriptor Pre-selection, a wide range of possible periods and numbers of periods were scanned, yet only these values were retained from a very large pool of candidate features, with the sole exception of a period of 10.0. Importantly, the selected descriptors also possess clear physical interpretations. It is worth noting that the exhaustive search over periodicity values (1.2–15.0 with a step of 0.1) did not capture any statistically justified descriptors corresponding to periodicities other than the expected values of 3.6, 3.0, and 10.0. This suggests that other periodicities do not have a significant influence on the formation of protein secondary structure.

We should mention KARS160108 (average weighted degree) [67]—the physicochemical amino acid property from the AAindex [68] database—which reflects the average connectivity of amino acids in a residue–residue interaction network according to the strength of each interaction. This measure is linked to the compactness and local packing density of protein structures, properties that are known to correlate with secondary structure elements such as *α*-helices and *β*-strands. Relatively high rank of KARS160108 among the selected descriptors suggests that network-based connectivity metrics capture structural constraints that cannot be represented explicitly by simpler physicochemical scales. The occurrence of properties reflecting free energy in the most frequently observed elements of protein secondary structure (*α*-helices and *β*-strands) confirms that free energy exerts the prominent influence on secondary structure formation.

Table 2 summarizes the periodic sequence properties associated with secondary structure that were identified using the descriptor pre-selection procedure (Section 4.5).

#### 2.1.3. Descriptors, Capturing PSS Non-Periodic Properties

For non-periodic descriptors, we observed a similar prevalence of physicochemical properties among the most important features. Table 3 lists all such descriptors among the top 109 identified via the descriptor pre-selection procedure (Section 4.5). Notably, some descriptors capture the impact on the structure at a given position while being computed over fragments that do not include that position itself.

#### 2.1.4. Results Obtained by the Combined Feature Set

The combined feature set results confirm that the proposed descriptor selection approach not only preserves biologically relevant periodic signals but also effectively reduces the dimensionality of the descriptor space and enhances prediction accuracy without interpretability loss.

In particular, the descriptor selection procedure retained a descriptor derived from IUPred2A [72], which estimates the intrinsic structural disorder directly from amino acid sequences. This alignment-free metric reflects the probability of a residue being located in a disordered or flexible region, often associated with domain boundaries or linker segments. The inclusion of IUPred2A output among the top-ranked features indicates that disorder-related information provides complementary structural signals that are highly relevant for protein secondary structure recognition, even in the absence of homologous sequences.

Unlike features derived from multiple sequence alignments (MSAs), both KARS160108 and the IUPred2A-derived descriptors encode structural organization solely from primary amino acid sequence information. Their presence among the top-ranked features supports the conclusion that alignment-free approaches can capture structural constraints that significantly improve prediction accuracy for proteins lacking close homologs.

We evaluated the performance of PSSP using the following ESM2 [8] models: esm2_t33_650M_UR50D (1280-dimensional embeddings, 33 layers, 650 million parameters) and esm2_t64_1B_UR50D (2560-dimensional embeddings, 64 layers, 1 billion parameters). These embeddings were employed as inputs to two- and four-layer bidirectional LSTM networks. As expected, the prediction accuracy with ESM2 embeddings alone was notably lower than that achieved by combining ESM2 embeddings with the selected descriptors described above. Specifically, the highest Q8 accuracy reached 79.71% for the ESM2-only configuration, compared to 84.46% when the selected descriptors were incorporated. This performance gain demonstrates that the selected physicochemical, periodicity-based, and alignment-free structural descriptors provide complementary information to the pre-computed ESM2 embeddings, resulting in a substantial improvement in the accuracy of protein secondary structure predictions. Comprehensive benchmarking results for all tested configurations are provided in Supplemental Table S8 (two-layer bi-LSTM network) and Supplemental Table S9 (four-layer bi-LSTM network).

### 2.2. Prediction Results

We report the significant performance advantage of our proposed method, TruMPET, over all previously published protein secondary structure prediction approaches at the time of submission—specifically those that operate without the use of evolutionary information or sequence alignments. For eight-class DSSP classification, TruMPET demonstrates a 10% improvement in accuracy compared to the nearest competing method, approaching the theoretical limit of PSSP in eight classes [74]. While TruMPET also outperforms existing methods in three-class DSSP prediction, the margin of improvement in this case is less remarkable.

The proposed TruMPET method comprises two models. The first, designated ‘LDA’, was defined by a single LDA analysis performed on a comprehensive non-redundant dataset (see Section 4.2 for details) and includes 109 descriptors (see Section 2.1, Section 2.1.1 and Section 2.1.2). The second improved model, referred to as ‘mix’, contains 583 descriptors and was obtained by combining the results of a few independent LDA analyses; the resulting features set was subsequently verified for mutual non-correlation using SDA. A detailed compilation of the ‘LDA’ and ‘mix’ datasets is provided in Section 4.1.

To benchmark our approach and compare it with the other existing state-of-the-art methods for predicting protein secondary structures, we selected the following widely applied non-redundant datasets: CB513 [75], TS115 [76], TEST2018 [77], TEST2020-HQ [78], CASP 13-FM [79] and CASP14-FM [80]. A detailed description of each dataset can be found in Section 4.2. A comprehensive discussion of benchmarking datasets and their impact on the assessment of evaluated methods can be found in [81]. The results of benchmarking the TruMPET method on the selected test datasets are presented in Table 4.

To compare the performance of our method, we selected the following renowned state-of-the-art protein secondary prediction methods and the most recent state-of-the-art methods found in the literature: SPIDER3-Single [82], MUFold-SS [83], ProteinUnet2 [84], SPOT-1D-Single [85], SPOT-1D-Profile and SPOT-1D-LM [78], MHTAPred-SS [27], ProtTrans [21], DML_SS^embed^ [86], and our dataset MilchStruct, consisting of mostly structural features, previously presented in [47]. All these methods, except for SPOT-1D-Profile, do not utilize sequence profiles or multiple sequence alignments (MSAs). To evaluate TruMPET’s predictive performance in comparison with these methods, all protein chains from benchmarking datasets were omitted from the assessment. The results of the comparison are presented in Table 5.

As can be seen, for the Q8 classification tasks, our method significantly outperformed both renowned state-of-the-art and recent secondary structure prediction methods reported in the literature, including our previous method and feature set (the values shown in Table 5).

Despite the substantial improvement in prediction accuracy for the eight-class DSSP, numerous applications still require secondary structure prediction within the conventional three-class representation. Therefore, a comparison of the accuracy of different PSSP methods under the three-class scheme is appropriate; the results of this comparison are presented in Table 6.

## 3. Discussion

In this study, we present an approach that combines ESM2 [8] embedding with two-step descriptor selection and a statistical testing procedure with the following employment of a two-layer bidirectional LSTM neural network.

### 3.1. Confusion Matrices and Their Analysis

**Figure 2:**
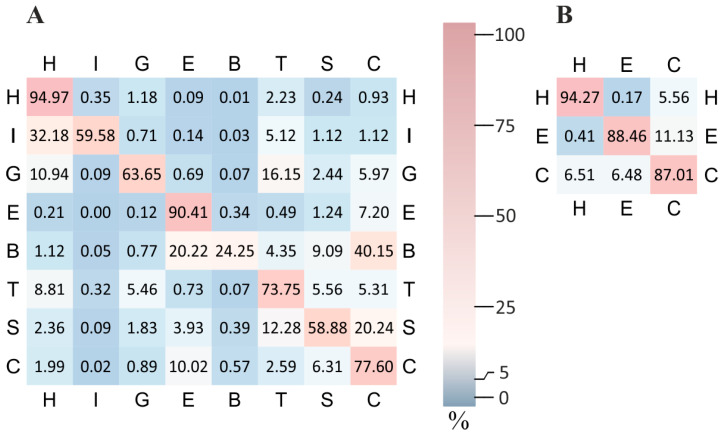
Normalized confusion matrices for eight-class (**A**) and three-class (**B**) secondary structure prediction obtained by ‘LDA’ model. Each row is normalized by the total number of true labels in that class. High precision is achieved for *α*-helix (H) and extended strands (E), while *β*-bridges (B) show noticeable confusion with other categories.

A detailed examination of the normalized confusion matrices (Figure 2) highlights the strengths and weaknesses of the proposed alignment-free model. The complete confusion matrices both for ‘LDA’ and ‘mix’ models can be found in Supplemental Table S3.

The highest predictive performance is observed for *α*-helix (H), a conformation basically determined by short-range interactions. Structurally related conformations such as *π*-helix (I) and 3_10_-helix (G) frequently produce false positives labels such as *α*-helix. Even hydrogen-bonded turns (T) are often confused with *α*- and 3_10_-helices, as reflected in the off-diagonal elements of the matrix. Bend (S) is frequently misclassified as either a turn (T) or a coil (C), consistent with the flexible and poorly defined nature of these conformations. The coil (C) class itself encompasses a broad range of structurally ambiguous or irregular states, making it a frequent fallback prediction. The prediction quality decreases notably for extended strands (E) and particularly for *β*-bridges (B). Both of these *β*-structure elements are strongly influenced by long-range interactions—either within a single polypeptide chain or between separate chains. Since the model is fully alignment-free and does not incorporate structural context beyond local residue features, it lacks the ability to take into account such interactions.

Taken together, these observations suggest that while our approach is appropriate for predicting secondary structure elements driven by local interactions, it remains challenged by *β*-structures that require modeling of long-range residue contacts. This limitation has important implications for the design of future high-accuracy prediction approaches. The accuracy of *β*-structure prediction may be enhanced either by incorporating alignment-based information or by employing protein inter-residue contact prediction strategies, such as those applied in ESM2 [8].

### 3.2. Importance of Descriptor Selection for PSSP

As we previously noted in [47], the choice of descriptor plays a decisive role in achieving a high prediction accuracy, whereas the neural network architecture primarily serves to unlock the potential embedded in the selected feature set. This conclusion is supported by the benchmarking results presented in this study; our method was compared to others that employ highly sophisticated neural architectures, such as those proposed in [27,31]. These methods utilize advanced designs, including the use of ESM2 embedding followed by Bi-LSTM layers—similar to the architecture employed in our approach. Nevertheless, their performance in the context of eight-class DSSP prediction is significantly lower than that of our method. These results suggest that future efforts in the field should prioritize the development of new, statistically grounded, and mutually uncorrelated descriptors sets, rather than focusing solely on refining already complex neural network architectures.

In particular, we confirmed that careful selection of descriptors is the primary determinant of model quality in the performed study. In our experiments, changes to the hyperparameters of the bi-LSTM architecture had only a minor effect on predictive performance, whereas the removal of any single high-rank descriptor category caused a substantial accuracy loss.

This study is the first to integrate non-canonical amino acids (ncAAs) into a deep learning-based secondary structure prediction framework. We employed previously determined physicochemical properties for both canonical and non-canonical amino acids; for frequently occurring non-canonical residues, we applied a one-hot encoding approach alongside physicochemical and contextual embedding features. Although physicochemical properties cannot be predicted for the most common [46] ncAA selenomethionine MSE ((2S)-2-amino-4-methylselanyl-butanoic acid), i.e., methionine modified by attaching a selenium atom, we employed MSE when compiling a list of potential descriptors by one-hot encoding—the 21 most frequently occurring non-canonical amino acids were added to the list of canonical amino acids.

The potential performance gain attributable to the integration of ncAAs into PSSP depends on the specific ncAA (some ncAAs are merely isomers of their canonical counterparts), the proportion of ncAAs within the protein (e.g., collagen and elastin contain a substantial fraction of hydroxyproline), and the extent to which the physicochemical and structural properties of the ncAA differ from its canonical counterpart. Accordingly, the incorporation of ncAAs in the prediction method can exert a significant impact on the PSSP accuracy for certain proteins. The PSSP results for a selection of proteins are presented in Table 7. The data demonstrate a notable enhancement in prediction accuracy when ncAAs are considered for these particular proteins.

An important distinction of our method is its alignment-free design: predictions are based solely on intrinsic physicochemical properties of amino acids and statistical patterns of local backbone conformation captured by the protein block (PB) formalism. While PBs can successfully encode local geometry, they do not capture long-range spatial interactions. The confusion matrix patterns observed in our results reflect this limitation, with a strong performance for *α*-helices and related helix types, but a reduced accuracy for *β*-structures.

These results are consistent with the known limitations of alignment-free approaches, which cannot exploit the evolutionary context captured by multiple sequence alignment (MSA). MSA-based methods such as NetSurfP-2.0 [87], SPOT-1D [77], and DeepCNF [88] partially overcome this challenge by implicitly capturing long-range interaction patterns through evolutionary profiles.

Among the selected features, KARS160108 (average weighted degree) [67] and the IUPred2A-derived [72] intrinsic disorder score can be considered as interpretable, alignment-free descriptors that capture the structural constraints implicitly represented in ESM2 embeddings. KARS160108 reflects network-based residue connectivity and local packing density, whereas IUPred2A provides information on structural flexibility and domain boundaries. Their presence among the top-ranked descriptors, as well as the observed accuracy gain when combined with ESM2 embeddings, suggests that such interpretable descriptors offer complementary information to large protein language models. This synergy suggests that the integration of alignment-free structural and physicochemical descriptors with pre-computed sequence-based LLM embedding represents a strategy for enhancing PSSP, particularly for proteins with limited or no detectable homologs in structural databases.

For assessing of the accuracy of prediction by three classes, it is evident that our method accurately discriminates H and E classes. This is expected, since in an RMSD-based approach, H and E classes have distinct RMSD separation (≈3.5Å for pentapeptides, see [89]). In other aspects, the properties of the three-class confusion matrix can be regarded as a simplified version of the corresponding eight-class matrix. The observed misclassification between H and C arises from the fact that the C class includes structural motifs such as turns, which are close to the H class by the RMSD. A similar effect explains the leakage from E to C: the C class also comprises conformations that are close to the E class by the RMSD (e.g., polyproline-II helix).

### 3.3. Comparative Evaluation of PSSP Accuracy with AlphaFold 2

Given that the known limitations of AlphaFold [9,24], discussed in [26], may have a limited impact on protein secondary structure, derived from AlphaFold’s predicted 3D structures, we performed a comparative assessment of AlphaFold 2 and TruMPET on Free Modeling targets from CASP14 [80], as well as proteins from [26] for which consistent data from PDB and AlphaFold are available. The results of this assessment are presented in Table 8.

As demonstrated in Table 8, advanced methods that leverage evolutionary information and MSAs (notably, AlphaFold 2) achieve higher Q8 accuracy when the entire target protein has a homolog with an experimentally resolved structure. The highest Q8 scores obtained by AlphaFold 2 correspond to cases in which it outperforms methods that do not use MSAs or evolutionary information, though the improvement is modest (typically one to two percentage points). In the intermediate Q8 range (0.6–0.8), alternative approaches frequently yield higher accuracy, whereas for Q8 values below 0.6, methods that do not rely on MSAs or evolutionary information generally perform better.

A thorough investigation of the predictions in Table 8 revealed that AlphaFold 2 often exhibits a substantial decline in prediction accuracy when the entire target protein sequence is homologous to a fragment of another protein that has a distinctly divergent experimentally resolved structure. This effect is particularly pronounced for small proteins, when the entire sequence of the small peptide is aligned to, and structurally interpreted as, a segment of a much larger homologous protein with a known structure. In such cases, AlphaFold 2 incorrectly predicts the structure of the small peptide as if it were part of the larger template. This limitation can be attributed to the fundamental design of AlphaFold, which relies heavily on MSAs and evolutionary relationships.

In contrast, alignment-free methods—specifically those that employ statistically validated, mutually uncorrelated structural and physicochemical descriptors (such as TruMPET) are inherently less susceptible to this artefact. This comparative accuracy evaluation supports the necessity and relevance of continued development of diverse alignment-free protein structure prediction approaches in the post-AlphaFold era.

Additionally, accurate alignment-free secondary-structure predictions provide explicit and interpretable local structural and physicochemical descriptors that can be used as input features in alignment-free 3D structure prediction pipelines, as well as in other downstream modelling tasks.

### 3.4. A Promising Avenue for Improving PSSP Accuracy

The discussion in Section 3.2 suggests that further improvements in the PSSP accuracy may be constrained by the archaic categorization of local conformations. As already discussed, elements assigned to class C are structurally close to the E class in Q3 representation, while the I (*π*-helix) and B (*β*-bridge) classes in Q8 are rare. Despite the fact that DSSP has been the de facto standard for secondary structure annotation for decades, its capacity is inherently limited by the original hydrogen-bond-based definition. The underrepresentation of classes B and I in protein structures gives rise to a severe class imbalance, which complicates both learning model generation and training. Concurrently, DSSP aggregates a wide array of heterogeneous conformations into the single class C (coil). Consequently, DSSP-based annotations (Q3/Q8) provide only a coarse-grained approximation of the local backbone geometry.

To achieve more detailed and physically meaningful representations, extended annotation schemes are necessary. One such approach is Protein Blocks [64,65], which discretize local conformations of pentapeptides into 16 structural states derived through clustering. Other structural classifications have also been proposed, differing not only in the number of discrete states but also in the fragment length used for discretization. Importantly, the choice of structural classification may depend on the specific context of the study, and its applicability for a given research (or even for a specific subtask) should be determined through numerical modeling.

### 3.5. Discussion of CASP FM Target Performance

Many methods exhibit a reduced predictive performance on FM categories in CASP. One plausible explanation is that this category includes proteins whose structures have been determined by NMR spectroscopy. It is well established that the structure of the same protein can differ substantially between NMR spectroscopy and X-ray crystallography [90]. Since our model, like many others, was trained primarily on X-ray-derived datasets, such discrepancies may contribute to performance degradation. Additional ambiguity arises from the necessity of selecting a single NMR model from the ensemble typically deposited in PDB/mmCIF files. Furthermore, in the case of X-ray data, some FM category targets in CASP correspond to structures resolved at a low resolution, which introduces additional imprecision into model evaluation.

### 3.6. Performance Gain Relative to the Previous Method

A comparison with our previous method [47] demonstrates a substantial improvement in the predictive accuracy achieved by the current method, TruMPET, with an increase of approximately 10% on the test datasets (Table 5 and Table 6). This enhancement can be attributed both to the implementation of a more sophisticated bi-LSTM neural network architecture and to the generation of the feature set through a substantially refined descriptor pre-selection procedure based on LDA.

## 4. Materials and Methods

### 4.1. Training Dataset Compilation

We retrieved a non-redundant set of protein chains from the PISCES server [91] using the following filtering criteria: sequence identity ≤ 40%, resolution ≤ 3.0 Å, sequence length between 40 and 10,000 residues, R-factor ≤ 0.3, and X-ray structures only. The dataset was generated on 7 July 2025, and initially contained 26,622 protein chains.

To prevent data leakage, we removed all chains present in the benchmarking datasets CB513 [75], TS115 [76], TEST2018 [77], and TEST2020-HQ [78], as well as targets from the free modeling category of CASP13 [77], CASP14 [80], and CASP15 [92] contests at the assessment analysis.

We randomly split this dataset into training and internal test subsets in a 4:1 ratio; 21,297 chains were employed for training and 5325 for internal validation. The internal test set was employed for hyperparameter tuning. Both datasets are available in Supplemental Table S4.

Structures for these datasets were downloaded from PDB [43] in mmCIF format.

### 4.2. Description of Benchmarking Datasets

To evaluate the classification performance of our new method, we also benchmarked it on the following widely used datasets:

- The CB513 [75] dataset, which remains a widely used benchmark that was designed specifically to evaluate the accuracy of secondary structure prediction methods. This dataset consists of 513 nonhomologous protein domains, accounting for 435 protein chains in total (some chains contain two or more domains). In this dataset, many protein chains are split into domains and are considered as separate targets. Our prediction method, however, accounts for the impact of each entire chain on every position. Indeed, the ESM2 embeddings we employ yield different representations for a fragment depending on the length of its parent chain. Therefore, we perform predictions using complete chains—that is why the “Number of Chains” in Table 1 is less than 513, although all CB513 segments are included in our performance evaluation.
- The TS115 [76] dataset, which contains proteins that were released after 1st January 2016 and whose structures were recovered via X-ray with a resolution ≤3.0 Å. Additionally, sequences with an identity >30% to those released before 2016 were removed. This dataset consists of 115 proteins.
- The TEST2018 [77] dataset, which consists of 250 proteins deposited between Jan 2018 and July 2018 with a resolution <2.5 Å and R-free < 0.25 that have sequence similarities of less than 25% to all pre-2018 proteins.
- The TEST2020-HQ [78] dataset, which includes all proteins released between May 2018 and April 2020, with the removal of homologues to all proteins released before 2018 on PDB [43]. Proteins with lengths greater than 1024 were also removed. Further constraints of <2.5 Å and R-free < 0.25 resulted in 124 proteins.

The CASP13 [79], CASP14 [80] and CASP15 [92] datasets, which represent targets from the free modeling category that contain proteins for which no known homologues existed at the time of the contests. In all CASP datasets, targets from both the free modeling (FM) category and the mixed free modeling/template-based modeling (FM/TMB) categories were included. A comprehensive discussion of benchmarking datasets and their impact on the assessment of evaluated methods can be found in [81].

### 4.3. Secondary Structures and Sequences Extraction

Secondary structure assignment was performed with mkdssp v.4.3.1 [23], available at https://github.com/PDB-REDO/dssp (accessed on 19 November 2025), which implements the DSSP algorithm [61] and is distributed as part of the PDB-related databanks [93].

Since we employed physicochemical properties from AAindexNC [46] for non-canonical amino acids as descriptors, we needed to extract both one-letter and three-letter amino acid sequences from the mmCIF files. In particular, the one-letter sequences were required to generate ESM2 embeddings. One- and three-letter sequences were extracted using an in-house Python 3.10 script, although at the time of publication, this could be achieved more easily with ProDy [94] v. 2.6.1, which supports convenient one-letter and three-letter sequence extraction from pdb/mmCIF formats.

### 4.4. Feature Set Compilation

The construction of a comprehensive set of descriptors is a crucial and challenging step in the data preparation process.

As the primary sequence representation, we employed embeddings derived from the ESM-2 protein language model (650 M parameters, trained on the UniRef50 dataset) [8]. For each amino acid residue, a 1280-dimensional vector was extracted from the final hidden layer of the model. ESM-2 embeddings have been shown to capture both the local sequence context and long-range dependencies, thereby providing an informative and alignment-free representation suitable for secondary structure prediction.

To improve predictions made by ESM-2, we developed two main types of additional descriptors. Descriptors generated by these types were added to the descriptors originating from ESM-2. The first type is based on the statistical occurrence of local structural fragments, more specifically protein blocks, within the protein chain. The second type relies on the physicochemical properties of amino acids originating from the AAindex database [66] and its extension, the AAindexNC database [46]. The approach for generating both types of descriptors has been described in detail in our previous works [47,55]. Briefly, instead of using raw physicochemical values, we applied a set of complex transformations intended to formulate our hypotheses regarding the determinants of secondary protein structure as accurately as possible. One of the most illustrative examples of this transformation is the generation of descriptors that can distinguish between *α*-helices and 3_10_-helices. These structural elements are known to be characterized by periodicities of approximately 3.6 and 3.0 residues, respectively. It is suggested that such helices tend to align themselves on the surface of a protein globule such that the hydrophobic side of the chain is directed inward, while the hydrophilic side is directed outward. This leads to a complex challenge involving the following:

- Selecting an appropriate physicochemical (e.g., hydrophobicity scale) or structural property across the many available properties;
- Quantitatively encoding the structural periodicity via a procedure that attenuates the impact of residues depending on their distance from the target position.

We addressed this using a specialized heuristic procedure, which is described in Section 4.5 Descriptor Pre-Selection. Specifically, we iteratively searched for the following:

- The optimal hydrophobicity scale (selected from all AAindex + AAindexNC properties);
- *T*—the periodicity described by this descriptor;
- The relevant weighting function that quantifies the attenuation of the contribution to the descriptor’s value with increasing sequence distance (in residues).

The periodic descriptor that formalizes our approach to encode the periodic properties of amino acid sequences into numerical descriptors can be written as:

(5) $$F\left(H_{n},\ldots ,H_{o},\ldots ,H_{n}\right)=\sqrt{{\left(\sum_{k=1}^{n}H_{k}\cos\left(k\frac{2\pi }{T}\right)\;f(k)\right)}^{2}+{\left(\sum_{k=1}^{n}H_{k}\sin\left(k\frac{2\pi }{T}\right)\;f(k)\right)}^{2}}$$

where *H_k_* is the value of a given physicochemical property from the AAindex database at position *k*; *T* is the period of the expected structural repeat; *f*(*k*) is the Gaussian-like decay function that captures the attenuation of the impact on the descriptor’s value at the current position as the residue-to-position distance increases:

(6) $$f(k)=\exp\left(-A\;{\left(\frac{k}{n_{T}}\right)}^{2}\right)$$

and $2n+1=T\;n_{T}$: 2*n* + 1 is the size of the analyzed window within the protein chain, i.e., the number of amino acid residues considered. This value is equal to the product of the period length *T* and the number of periods $\;n_{T}$. Thus, to identify descriptors that reflect the periodicities inherent in the backbone structure, we systematically tested all reasonable combinations of the parameters *T*, $\;n_{T}$, and *A* across the full set of 566 physicochemical properties available in the AAindex database, e.g., to capture both short 3_10_-helices and longer *α*-helices, as well as other potential implicit periodic patterns, we tested *T* in the range of 1.2 to 15.0 with a step size of 0.1, $\;n_{T}$ from 2 to 9 with a step of 1, and *A* was varied over the values 0.5, 1, 2, and 3.

To design descriptors that capture non-periodic physicochemical properties, we employed simpler features reflecting the aggregated physicochemical characteristics of sequence fragments. These descriptors were generated by summing the values of a given physicochemical property from the AAindex database across a predefined window of amino acid residues, using a Gaussian-like decay function similar to the one described earlier. We evaluated all properties from the AAindex database, and for each physicochemical property, we varied the start and end positions of the fragment relative to the target residue (position 0), e.g., we tested whether the cumulative value of a property from positions −10 to −3 correlates with the observed conformation at position 0. A detailed description of all feature types is provided in Supplement S2.

A similar approach was applied for the generation of descriptors based on the statistical occurrence of local structural fragments. In this context, local fragments refer to the set of 16 protein blocks defined by de Brevern et al. [62]. These 16 five-residue fragments form the basis of a generalized structural alphabet, providing a more flexible description of the local backbone conformation than traditional secondary structure classification. As shown in our earlier work [89], there exists a mutually unambiguous correspondence between protein blocks and Cartesian coordinates, enabling a compact and informative encoding of the local backbone geometry. The conformation of any pentapeptide can be represented by a vector of 16 RMSD values, each corresponding to a deviation from one of the 16 reference protein blocks. Feature generation is then based on statistical assessment of the hypothesized relationships between these values and the underlying amino acid sequence. As in the case of physicochemical properties, this process involves the combinatorial tuning of multiple parameters that define the characteristics/specificity of these relationships. The number of possible candidate descriptors is extremely large—especially in the present study, where we employed a much broader and more diverse set of parameterizations than in our previous works. To manage this complexity, we developed a dedicated optimization procedure, which is described in detail in Section 4.5 Descriptor Pre-Selection.

### 4.5. Descriptor Pre-Selection Procedure

As we previously described [55], a complete set of features must consist of statistically significant, mutually uncorrelated descriptors that reflect the fundamental principles that define the protein structure that will be predicted.

To optimize the set of input features, we employed a two-step selection approach. In the first step, we applied a fast Stepwise Discriminant Analysis (SDA) [56] to reduce the initial descriptor space while retaining a subset of mutually uncorrelated descriptors. In particular, we tuned the parameters for the descriptors defined by Equations (4) and (5), as well as for other types of descriptors, including those derived from the AAindex database. By employing this pre-selection step, we efficiently reduced the vast pool of potential descriptors by approximately two orders of magnitude (from 10,000 to ~500) before applying more advanced modeling techniques. This procedure not only reduced the computational resource requirements, but, more importantly, also retained only statistically significant and mutually uncorrelated descriptors in the final feature set.

In the second step, we applied a more computationally intensive ‘greedy’ algorithm for Linear Discriminant Analysis (LDA) [62] implemented with parallel processing to enhance computational efficiency. This step was essential for boosting the classification accuracy from 50.5% to 64.6% (see Section 2). An additional motivation for introducing the SDA prior to LDA was the substantial reduction in descriptors—from 10,000 to ~500—that made subsequent LDA calculations considerably more tractable. Together, this computational and accuracy gap clearly justified the implementation of a two-stage pre-selection strategy for feature set generation.

At each step of the ‘greedy’ procedure, the descriptor that demonstrated the greatest improvement in accuracy was added to the features set. Although not exhaustive, this strategy represents a ‘greedy’ approximation to best-subset selection [95], as it evaluates all remaining descriptors in the context of those already selected. This process continued until either no further improvement above a minimal threshold was observed or a predefined accuracy target was achieved. The result of this analysis is ‘LDA’ model that we reported in Section 2.

The scripts that implement SDA and LDA and all descriptors (both initial and processed) are freely available at the following Github page: https://github.com/Milchevskiy/TruMPET.2025 (accessed on 19 November 2025). The description of the software implementation and the usage of the Descriptors Pre-Selection Procedure are provided in Supplement S4.

For further improvement, this procedure can be iteratively repeated, guided by various hypotheses regarding factors influencing protein secondary structure formation. The resulting descriptor sets are then merged, with exact duplicates removed, and subjected to an additional SDA analysis. This process yields the combined ‘mix’ model reported in Section 2. The full list of descriptors constituting the ‘mix‘ model is provided in Supplementary Table S6.

### 4.6. Neural Network Architecture

For ‘LDA’ model we employed a two-layer bidirectional LSTM (hidden unit size 512 per direction, with dropout initially set to 0.7 and gradually reduced to 0.0 once the validation accuracy plateaued), followed by a three-layer feed-forward classification head (2048→1024→512→9 with the ReLU activation function). The output layer comprises the standard eight DSSP classes and an additional ninth technical class (Ø), specifically introduced to handle regions of unresolved structure in protein chains. In such cases, no structural information is available at certain positions, yet periodic or long-range descriptors may still exert influence across these regions. This technical class does not represent a biological category of secondary structure and was not involved in model fitting. All such positions were labeled as ‘ignore’ in the loss function (CrossEntropyLoss with ignore_index) and were excluded from the calculation of all evaluation metrics. Thus, training and evaluation were performed strictly on the eight valid DSSP classes, while the ninth class served solely as a mask for missing data. To efficiently handle variable-length protein chains within a mini-batch, sequences were processed as PyTorch (version 2.9.1) PackedSequences, and padding was ignored in loss/metrics via ignore_index. Training was achieved using the Adam (initial lr = 1 × 10^−4^, weight decay = 1 × 10^−4^) optimizer, with a stepwise reduction in the learning rate down to 1 × 10^−5^ after dropout was annealed to 0. Early stopping (patience = 13) was applied. The residue-level accuracy and macro-F1, along with a confusion matrix, were evaluated. The best checkpoints were saved for both GPU and CPU, and all training logs and curves were automatically recorded to ensure reproducibility.

In addition, we performed a systematic exploration of hyperparameters, including the number of LSTM layers (2–4), hidden sizes (256–1024), dropout schedules, and learning rate decay strategies. We also tested different batch sizes and optimizer configurations. The experiments consistently demonstrated that deeper bidirectional architectures with adaptive dropout and learning rate schedules led to the best trade-off between predictive accuracy and training stability, justifying their use in the final model.

**Figure 3:**
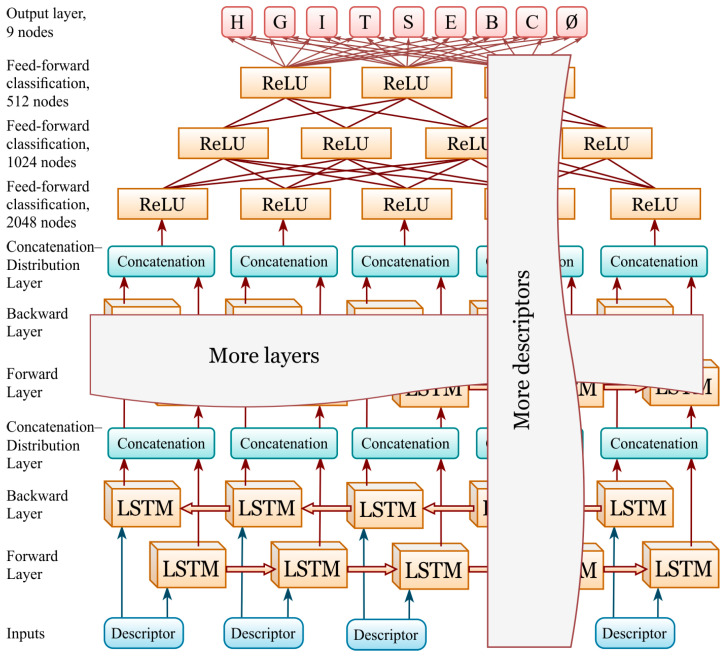
Neural network diagram: four-layer bidirectional LSTM (hidden size 1024 per direction, dropout initially set to 0.7 and gradually reduced to 0.0 as the validation accuracy plateaued) followed by a three-layer feed-forward classification head (2048→1024→512→9 with the ReLU activation function). The output layer includes 8 DSSP classes and an additional 9^th^ class (Ø) introduced to handle regions of unresolved structure in protein chains.

For the ‘mix’ model, the hyperparameters are identical to those of the ‘LDA’ model, except for the number of bi-LSTM layers (four) and the hidden unit size per direction (1024). Figure 3 presents schematic representation of the neural network used in this study.

The scripts that implement both models learning are freely available at the following Github page: https://github.com/Milchevskiy/TruMPET.2025 (accessed on 19 November 2025). The description of scripts for neural network training is provided in Supplement S7.

### 4.7. Prediction Quality Evaluation Metrics

In this work, we employed the most widely used measure for evaluating PSSP performance—the Q measure, defined as the percentage of correctly predicted residues. Originally formulated in [96], for three DSSP classes, it can be formulated as

(7) $$Q3=\sum_{S\in \{C,H,E\}}\frac{O_{+}(S)}{N}$$

where *O*_+_(*S*) is the number of correctly predicted residues in class *S* and *N* is the total number of residues in the query protein. Importantly, this measure is independent of the number of prediction classes and can therefore be applied consistently to three-class, 8-class, or even 16-class (protein block) prediction tasks.

The traditional F-measure, or balanced F_1_ score [97], is an additional ML evaluation metric that assesses a model’s predictive performance on a per-class basis, rather than providing an overall accuracy measure. It combines precision and recall through their harmonic mean, such that maximizing the F_1_ score requires simultaneously maximizing both precision and recall. The F_1_ score ranges from 0 (worst) to 1 (best) and can be expressed as:

(8) $$F_{1}=\frac{2\times \mathrm{TP}}{2\times \mathrm{TP}+\mathrm{FP}+\mathrm{FN}}$$

where TP is the number of true positives, FP is the number of false positives and FN is the number of false negatives.

In addition, confusion matrices [98] were applied to provide extended information about the interrelationships among predicted classes of secondary structures. In this study, each row corresponds to the true class, whereas each column corresponds to the predicted class. This allows for a detailed analysis of misclassifications, i.e., cases where the model confuses one structural class with another.

## 5. Conclusions

The presented protein secondary structure prediction method, TruMPET, operates without any reliance on evolutionary information or structural data from homologous proteins and supports the procession of non-canonical amino acids. This framework is particularly advantageous for predicting the structure of proteins that lack homologs with experimentally determined structures or contain substantial proportions of non-canonical amino acids residues that affect their structural or physicochemical properties.

The problem of predicting, rather than merely recognizing, protein secondary structure cannot be regarded as solved, despite the near-theoretical performance achieved by the most advanced methods. As demonstrated in this manuscript, the results obtained by even state-of-the-art language models such as ESM2 can be further improved, primarily through extending their standard embedding feature sets with physicochemical and structural descriptors that capture the fundamental principles underlying the formation of the protein secondary structure. This enhancement is much more effective than increasing the architectural complexity of the neural network, which is not directly beneficial.

## Figures and Tables

**Table 1:** Top 15 most statistically significant RMSD-based descriptors.

LDA Index	Alphabet	Reduced Alphabet Length	PB Sequence	Offset	Power
1	Full	5	ACDDDFB	0	1
2	Full	5	PAP	0	1
3	Full	5	EHJIA	0	1
4	Full	5	MMMMM	5	1
7	PB_W7_tail_GP	7	MMMMMMM	0	1
11	Full	5	MMMMM	−4	1
14	Full	5	PAFKL	0	1
16	Full	5	DDDDD	0	1
17	Full	5	KLMMM	2	1
22	PB_w11_tail	11	PGB	0	2
24	PB_w11_tail	11	JIA	0	1
30	PB_W3	3	ACDDDFB	0	1
31	Full	5	DDFBG	0	1
34	Full	5	GHI	0	1
36	Full	5	KLN	−3	2

LDA index denotes the influence degree of physicochemical or structural descriptor to the protein backbone configuration prediction—the lower the index value, the higher its impact on the prediction accuracy; offset—the displacement from the central position of the PB fragment; alphabet—full (20 canonical amino acids) or reduced alphabet, see Supplement S2.

**Table 2:** Statistically significant physicochemical properties from the AAindex database associated with specific periodicities of protein secondary structures.

LDA Index	Property	Period	Number of Periods	Power	Description
25	KARS160108	3.7	5	1	Average weighted degree [67]
51	MUNV940103	10.0	3	2	Free energy in *β*-strand conformation [69]
63	MIYS990102	3.6	7	1	Optimized relative partition energies—method A [70]
77	MUNV940102	3.0	2	1	Free energy in α-helical region [69]

LDA index denotes the influence degree the physicochemical or structural descriptor has on the protein backbone configuration prediction—the lower the index value, the higher its impact on the prediction accuracy. Number of periods denotes the number of consecutive periods associated with the appropriate property.

**Table 3:** Statistically significant non-periodic physicochemical and structural properties contributing to the protein backbone conformation.

LDA Index	Property	Left Offset	Right Offset	Power	Description
6	MUNV940103	−5	6	1	Free energy in *β*-strand conformation [69]
8	PTIO830101	−5	6	1	Helix–coil equilibrium constant [71]
12	IUPred2 short mode			3	IUPred2 [72] predicts intrinsically disordered regions and domain boundaries
21	MUNV940102	−5	16	3	Free energy in *α*-helical region [69]
35	PTIO830101	−15	16	3	Helix–coil equilibrium constant [71]
60	PONP800104	−5	5	2	Surrounding hydrophobicity in *α*-helix [73]
73	End_N_3			3	The current amino acid position is ≤3 residues from the N-end
87	PTIO830101	−5	5	3	Helix–coil equilibrium constant [71]
93	MUNV940103	−5	5	2	Free energy in *β*-strand conformation [69]

LDA index denotes the influence degree of physicochemical or structural descriptor to the protein backbone configuration prediction—the lower the index value, the higher its impact on the prediction accuracy. Left and right offset—the distances from the current residue position within the amino acid sequence.

**Table 4:** Results of benchmarking the TruMPET method on various test datasets.

Dataset	Q8, LDA	F_1:8_, LDA	Q3, LDA	F_1:3_, LDA	Q8, Mix	F_1:8_, Mix	Q3, Mix	F_1:3_, Mix	Dataset Size, Chains
Validation	0.8343	0.6949	0.9015	0.8992	0.8446	0.7137	0.9085	0.9062	5325
CB513	0.8557	0.7461	0.9148	0.9128	0.8624	0.7599	0.9175	0.9157	435 *
TS115	0.8548	0.7411	0.9153	0.9096	0.8647	0.7628	0.9175	0.9119	115
TEST2018	0.8541	0.7518	0.9149	0.9122	0.8624	0.7671	0.9178	0.9150	245
TEST2020-HQ	0.8253	0.6882	0.8877	0.8841	0.8378	0.7091	0.8949	0.8911	124
CASP13	0.7876	0.6456	0.8700	0.8683	0.7957	0.6619	0.8704	0.8684	40
CASP14	0.7461	0.5438	0.8475	0.8472	0.7646	0.5668	0.8522	0.8513	34
CASP15	0.7286	0.5600	0.8338	0.8366	0.7357	0.5674	0.8402	0.8426	39

* In the CB513 dataset, many protein chains are split into domains and are considered as separate targets. Our prediction method, however, predicts on complete chains; thus, the chain number is less than 513, although all CB513 segments are considered (see Section 4.2). The process of compiling the ‘LDA’ and ‘mix’ datasets is described in Section 4.1. The table contains the results obtained from the complete chains list for each dataset.

**Table 5:** Results of benchmarking of various methods on eight DSSP classes.

Method	CB513	TS115	TEST 2018	TEST 2020-HQ	CASP13-FM	CASP14-FM
TruMPET, LDA	0.8541	0.8219	0.8417	0.8103	0.7712	0.7281
TruMPET, mix	**0.8608**	**0.8352**	**0.8486**	**0.8179**	**0.7799**	**0.7488**
MilchStruct	0.7940	0.7491	0.7606	0.7537	0.6207	0.5140
SPIDER3-Single	0.5464	0.5965	0.5981	0.5823	0.6081	0.5914
MUFOLD-SS	0.7432	0.6257	0.7429	—	0.7185	0.6863
ProteinUnet2	—	—	0.7460	0.5878	0.6081	—
SPOT-1D-Single	0.5464	0.5965	0.7217	0.6035	0.6093	0.6177
SPOT-1D-Profile	—	—	0.7541	0.7041	0.7122	0.6186
SPOT-1D-LM	0.6489	0.6832	0.7647	0.6773	0.7095	0.6318
MHTAPred-SS	0.7653	—	0.7728	—	0.7600	0.7254
ProtTrans	0.7450	0.7710	—	—	—	—
DML_SS^embed^	0.7554	—	0.7648	—	0.7417	0.7022

Bold indicates the best results within the table. All methods, except for SPOT-1D-Profile, do not utilize sequence profiles or multiple sequence alignments (MSAs). Protein chains present in the benchmarking datasets were excluded from the evaluation process of TruMPET. ‘—’ indicates that the corresponding result is not reported in the literature or could not be computed. A comprehensive discussion of benchmarking datasets and their impact on the assessment of evaluated methods can be found in [79].

**Table 6:** Results of benchmarking various methods on three DSSP classes.

Method	CB513	TS115	TEST 2018	TEST 2020-HQ	CASP13-FM	CASP14-FM
TruMPET, LDA	0.9136	0.8970	0.9064	0.8787	0.8595	0.8364
TruMPET, mix	**0.9166**	**0.8993**	**0.9088**	**0.8824**	0.8585	**0.8420**
MilchStruct	0.8599	0.8254	0.8336	0.8220	0.7183	0.6524
SPIDER3-Single	0.7367	0.7556	0.7257	0.7102	0.7512	0.7188
MUFOLD-SS	0.8444	—	0.8463	—	0.8343	0.7872
ProteinUnet2	—	—	0.7257	0.7128	0.7439	—
SPOT-1D-Single	0.7367	0.7556	0.7428	0.7222	0.7321	0.7470
SPOT-1D-Profile	—	—	0.8618	0.8197	0.8355	0.7566
SPOT-1D-LM	0.8841	0.8786	0.8674	0.7970	0.8215	0.7654
MHTAPred-SS	0.8743	—	0.8742	—	**0.8604**	0.8321
ProtTrans	0.8620	0.8690	—	—	—	—
DML_SS^embed^	0.8641	—	0.8682	—	0.8495	0.8075

Bold font indicates the best results within the table. All methods, except for SPOT-1D-Profile, do not utilize sequence profiles or multiple sequence alignments (MSAs). Protein chains present in the benchmarking datasets were excluded from the evaluation process of TruMPET. ‘—’ indicates that the corresponding result is not reported in the literature or could not be computed. A comprehensive discussion of benchmarking datasets and their impact on the assessment of evaluated methods can be found in [79].

**Table 7:** Accuracy gain for specific protein chains obtained by incorporating ncAA into TruMPET.

Protein Chain	Q8-LDA, ncAA	ncAA	ncAA Amount	Q8-LDA, AA	AA	Accuracy Gain, %
1P9GA	0.9024	PCA	1	0.7805	GLU	12.19
4XA6A	0.8036	MLY	16	0.7202	LYS	8.34
3EJHE	0.8000	HYP	3	0.7333	PRO	6.67
4WIDB	0.8711	MLY, MLZ	6	0.8338	LYS	3.73
7RUPA	0.9206	CSO	1	0.8889	CYS	3.17
5BQ8A	0.7885	MLY	4	0.7596	LYS	2.89
3UFIA	0.8237	MLY	14	0.7986	LYS	2.51
4QE0B	0.8323	MLY	16	0.8084	LYS	2.39
4QE0A	0.8708	MLY	16	0.8483	LYS	2.25
2VGXB	0.9172	MLY	9	0.8966	LYS	2.06
3KV0A	0.9381	MLY	17	0.9175	LYS	2.06
6S98A	0.8986	OCS	1	0.8784	CYS	2.02

ncAA—non-canonic amino acid. AA—counterpart of specific ncAA, canonic amino acid.

**Table 8:** Comparative assessment of PSSP accuracy for selected protein chains predicted by AlphaFold2 and TruMPET ‘mix’ model.

Protein Chain	AlphaFold2 Model	Q8, AlphaFold 2	Q8, TruMPET	Accuracy Remainder, %
CASP14 [78] Free Modeling Category Targets				
7D2OA	T1027TS427_1-D1	0.5906	0.6250	+3.44
6VR4A	T1040TS427_1-D1	0.7538	0.7124	−4.14
7BGLHXX	T1047s2TS427_1	0.6706	0.7026	+3.20
7ZHJEA	T1061TS427_1-D1	0.4844	0.5880	+10.36
7REJA	T1070TS427_1-D1	0.8026	0.8179	+1.53
7UM1A	T1096TS427_1-D2	0.8246	0.8035	−2.11
Proteins from [26]				
1S4TA	AF-P23907-F1-model_v6	0.2381	0.3333	+ 9.52
2KQPA	AF-P01308-F1-model_v6	0.4651	0.5465	+ 8.14
6OFSA	AF-P31828-F1-model_v6	0.9171	0.8564	−6.07
3T5OA	AF-P13671-F1-model_v6	0.7026	0.7061	+0.35
1WVKA	AF-O64818-F1-model_v6	0.3718	0.4615	+8.97
4ORWA	AF-P41222-F1-model_v6	0.9623	0.9497	−1.26

AlphaFold2 predicted structures for CASP14 were obtained from the official CASP server https://predictioncenter.org/casp14/results.cgi?view=targets&model=first&groups_id=205&tr_type=all&dm_class=fm (accessed on 19 November 2025) and for the proteome proteins—from the official AlphaFold server—for insulin (2KQP), it is https://alphafold.ebi.ac.uk/entry/P01308 (accessed on 19 November 2025).

## Data Availability

The source code for all scripts used in this work, the final learning model, and the detailed descriptions how to run training process are available at GitHub https://github.com/Milchevskiy/TruMPET.2025 (accessed on 19 November 2025).

## Supplementary Materials

The following supporting information can be downloaded at: https://www.mdpi.com/article/10.3390/ijms262311284/s1.

## Abbreviations

The following abbreviations are used in this manuscript:

DSSP	Dictionary of Secondary Structure in Proteins
PSSP	Protein Secondary Structure Prediction
PSS	Protein Secondary Structure
SDA	Stepwise Discriminant Analysis
LDA	Linear Discriminant Analysis
ML	Machine Learning
ncAA	Non-canonical Amino Acid
