# Supplementary material for: TruMPET: A New Method for Protein Secondary Structure Prediction Using Neural Networks Trained on Multiple Pre-Selected Physicochemical and Structural Features
Source: Int J Mol Sci. 2025 Nov 21;26(23):11284. doi: 10.3390/ijms262311284 (PMC12692721; doi:10.3390/ijms262311284)
Supplement: Supplementary file 1 [file ijms-26-11284-s001.zip › Supplement S2.Descriptors.Generation.pdf]

**Supplement S2. Descriptors Generation Description**

**Databases.**

Database of physicochemical properties

Database `Databases/aaindex/aaindex_mutant3.txt` contains physicochemical properties from the AAindex [66] and AAindexNC [46] databases for canonical and non-canonical amino acids. Amino acid names are given in the three-letter code. Only amino acids for which physicochemical properties could be evaluated [46] are included. Non-canonical amino acids containing atoms not presented in canonical amino acids (e.g., F, Cl, Ag, Se…) are not represented in this database.
A fragment of this database is shown below. The first row lists amino acid names. Subsequent rows contain the names of physicochemical properties (as defined in the AAindex database) and their corresponding values for each amino acid.

| Property | ALA | CYS | ASP | … | TY2 | TYS | XYG |
|---|---|---|---|---|---|---|---|
| ANDN920101 | 4.350 | 4.650 | 4.760 | … | 4.63029 | 5.86997 | 4.39422 |
| ARGP820101 | 0.610 | 1.070 | 0.460 | … | 3.02192 | -0.25478 | 0.78183 |

Databases of Amino Acid Sequence Fragment Frequencies in Protein Block Conformations

Databases containing frequency statistics of various protein block conformations are located in the directory `Databases/FrequencyExtrapolation`. In total, there are 22 such databases, each stored in its own directory, which also serves as its identifier. The databases differ in the length of the protein chain fragments and in the method of reducing the alphabet of 20 standard amino acids. Below you can see the example of `current.degeneration` file for `Databases/FrequencyExtrapolation/PB_W7_tail_GP` database:

```
//
-3   A V L I P M C F Y W K R H D E N Q S T G O X
-2   A V L I P M C F Y W K R H D E N Q S T G O X
-1   ALMC VIFYWKRHDENQST GP O X
 0   ALMC VIFYWKRHDENQST GP O X
 1   ALMC VIFYWKRHDENQST GP O X
 2   A V L I P M C F Y W K R H D E N Q S T G O X
 3   A V L I P M C F Y W K R H D E N Q S T G O X
```

The first column is the relative chain position. At positions −3 and −2 all amino acids are different. At position −1, ALMC amino acids are indistinguishable, as are VIFYWKRHDENQST, etc. With the above degeneration, the following fragments of the sequence:

```
AAAAAAA
AALLLMC
AALMCAA
```

are indistinguishable.
The database contains the number of such indistinguishable fragments, the average RMSD of each PB and the corresponding standard deviations. For each database, the type of degeneration and the size of the current window were specified in file `current.degeneration` file. In particular, PB_W7_tail_GP database has window size = 7.

Databases availability

Precomputed databases are available from our server (7.5 GB) as the archive:
https://ftp.eimb.ru/Milch/TruMPET.2025/Databases/TruMPET2025.databases.tar.xz
Unpack this archive into the Databases directory. Alternatively, you can recompute databases directly from the PDB [43] databank following the short guide:
https://ftp.eimb.ru/Milch/Generate.DB.Descriptors/readme.txt
or the full manual:
https://ftp.eimb.ru/Milch/Generate.DB.Descriptors/Generate.Databases.and.Descriptors.pdf

When using the manuals, you can stop after Step 2: MakeFrequencyDatabases if your purpose is to generate the frequency databases.

**Features generation**

1. <u>Physicochemical amino acids properties based descriptors.</u>

There are two types of functional transformations that are implemented right now: _Dull_Sum_tail (the sum of current property values in the window), c_Fourier_Smoothed (periodicity search for the current property with the smoothing by bell-shape function). Amino acid sequence is mapping to an array, corresponding to the selected property from the AAindex [66] database. *E.g.*, for the amino acid sequence AGP and the property PTIO830101 (helix-coil equilibrium constant, https://www.genome.jp/dbget-bin/www_bget?aaindex:PTIO830101) [69], the corresponding array would be {1.10, 0.60, 0.10}, according to the values from the following table:

| A/L | R/K | N/M | D/F | C/P | Q/S | E/T | G/W | H/Y | I/V |
|---|---|---|---|---|---|---|---|---|---|
| 1.10 | 0.95 | 0.80 | 0.65 | 0.95 | 1.00 | 1.00 | 0.60 | 0.85 | 1.10 |
| 1.25 | 1.00 | 1.15 | 1.10 | 0.10 | 0.75 | 0.75 | 1.10 | 1.10 | 0.95 |

As noted in the Databases section of this Supplement, we extended AAindex with physicochemical properties of non-canonical amino acids for which such predictions are possible [46]. Consequently, in the present work, physicochemical properties are associated with the three-letter amino acid codes, allowing the direct incorporation of non-canonical amino acids that are present in widely distributed proteins such as collagen.
Further, a functional transformation of this descriptor is performing according to the rules that are determined by a configuration line for this descriptor in the control file. *E.g.*

```
Dull_Sum    CHOP780208  0     15    1
```

`Dull_Sum` Type of transformation function (mandatory item)
`CHOP780208` Physicochemical property from AAINDEX database
`0 15` window start and end relatively to the current position in the protein backbone chain
`1` the power of descriptor (linear, quadratic, square root, etc).

This descriptor calculates the sum of the properties corresponding to CHOP780208 (normalized frequency of N-terminal beta-sheet, https://www.genome.jp/dbget-bin/www_bget?aaindex:CHOP780208 [97]) within the amino acid sequence, ranging from position 0 (the current position in the protein backbone chain) to 15. The final value is determined by raising the sum to a power, which can be any decimal value.
The initial construction of preliminary prediction models relies on an extensive set of input descriptors. This set is derived using various transformation functions with different parameters. The stepwise regression analysis method, employed in constructing these preliminary models, is capable of selecting statistically significant descriptors. This helps in identifying the most appropriate transformation functions and their corresponding parameters.
Descriptors serve to formalize our assumptions about the factors influencing the protein's structure. For instance, if we assume that the property PTIO830101 is significant within the range of -5 to 5, the descriptor will take the following form:

```
_Dull_Sum_tail          PTIO830101  -5    5     1
```

If we presume that the relationship may be nonlinear, it makes sense to include the following descriptors in the model:

```
_Dull_Sum_tail          PTIO830101  -5    5     2
_Dull_Sum_tail          PTIO830101  -5    5     3
_Dull_Sum_tail          PTIO830101  -5    5     0.5
```

The example of more complex descriptor:

```
c_FourierSmoothed       WERD780101  3.6   3     1
```

The functional transformation of this descriptor reflects a periodic change in hydrophobicity with a period of T=3.6. The parameter "3" characterizes the diminishing influence as you move away from the current position. It's a smoothing parameter, where a larger value implies stronger smoothing. You can find detailed implementation information in the file `CowardVariables/c_FourierSmoothed.cpp`. The value of this

descriptor reach maximum when the variation of the WERD780101 property (propensity to be buried inside, https://www.genome.jp/dbget-bin/www_bget?aaindex:WERD780101, [98] ) along the chain corresponds to a period of 3.6. The mass generation of descriptors for this transformation function involves testing a range of suitable properties from AAindex, along with all other relevant parameters:

```
_c_FourierSmoothed      WERD780101  1.2   3     1
…
_c_FourierSmoothed      WERD780101  3.6   3     1
…
_c_FourierSmoothed      PTIO830101  3.6   3     1
_c_FourierSmoothed      PTIO830101  1.2   3     1
_c_FourierSmoothed      PTIO830101  1.2   4     1
_c_FourierSmoothed      PTIO830101  1.2   4     2
```

2. <u>RMSD structure-based descriptors.</u>

There are 4 types of structure-based descriptors. First three types of descriptors are based upon t-test comparing the means of two independent samples. Let us consider one of the sixteen protein blocks $PB_j, j \in \{1, 2, 3, \ldots, 16\}$, and a 5-residue sequence *seq*. Also, let $N_{occ}(seq)$ be the number of times sequence *seq* occurs among the sequences with known structures (the training sample), $\bar{\mu}_j = \bar{\mu}_j(seq)$ be the mean distance between the structures with that sequence and the $PB_j$. Further, let $\bar{\mu}_j$ be the average distance between $PB_j$ and all 5-residue fragments in the training sample, and $s_j^2$ be its sampling variance and $N$ to be the size of the training sample. Then, according to Student t-statistics, one example of the descriptors is

$$t_j(seq) = \frac{\bar{\mu}_j - \bar{\mu}_j(seq)}{\sqrt{s_j(seq)}}, \text{ where } s_j(seq) = \frac{\sigma_j^2(seq)}{N_{occ}(seq)} + \frac{s_j^2}{N}$$

Note that if $N \gg N_{occ}(seq) > 1, \quad s_j(seq) \cong \frac{\sigma_j^2(seq)}{N_{occ}(seq)}$, the following folds to:

$$t_j(seq) \cong \frac{\bar{\mu}_j - \bar{\mu}_j(seq)}{\sigma_j(seq)} \sqrt{N_{occ}(seq)} \qquad \text{(S1)}$$

Thus, the number of occurrences of as certain sequence in the sample, $N_{occ}(seq)$ is crucial for correct estimation of $t_j(seq)$. Namely, small values of $N_{occ}(seq)$ may yield unreliable estimates of $\sigma_j(seq)$ as well as $t_j(seq)$. To alleviate this, we tried various reduced alphabets [87].

a. `_Student_emasculate` T-statistics value, calculated by formula (1)
b. `_T_statistics_single` Probability by t-test statistics, calculated by formula (1)
c. `_T_statistics_window` Sum the t-test probabilities in the window
d. `_Log_occurence_differrence` Occurrence difference, calculated by formula

$$value = \log(1 + \log(1 + N_{occ})) * (\mu_j - \bar{\mu}_j(seq))$$

Each descriptor can be both for direct and inverse RMSDs. In the case of the inverse RMSDs descriptor name concatenates with the _inv suffix. E.g. coupled to `T_statistics_window` descriptor for the inverse RMSDs has the name `T_statistics_window_inv` with the same parameters. There are 11 ready-to-use RMSD databases, any database can be applied to any descriptor. Parameters of each database can be found in the '`current.degeneration`' and '`sheduler`' files of the appropriate subdirectory of `Databases/Frequency_extrapolation/` directory.

a. `PB_w11_tail` Window size = 11, parameters to find $\beta$-turn
b. `PB_w11_tail_GP` Window size = 11, parameters to find $\beta$-turn, P and G are identical in the range [−2, 2]
c. `PB_W4C` Window size = 5, parameters to find $\alpha$-helix ends
d. `PB_W4N` Window size = 5, parameters to find $\alpha$-helix
e. `PB_W5_noDEG_ZIP` Window size = 5, no degeneration at all
f. `PB_W5_noDEG_ZIP_denom` Window size = 5, no degeneration at all, optimized for reverse RMSD

g. `PB_W6_3_trivial_PG` Window size = 6, coordinates [−2, 3], classification by amino acids classes (aliphatic, polar, acidic, basic, unique). Origin in the window is in '2' position.
h. `PB_W6_4_trivial_PG` The same as above, but origin in the window is in '3' position.
i. `PB_W7_tail_GP` Window size = 7, parameters to find turn with the possible interaction at window's ends.
j. `PB_W7_trivial_PG` Window size = 7, classification by amino acids classes (as in 'g').
k. `PB_w9_tail` Window size = 9, parameters to find any turn at longer window size.

Examples of complete records for structure-based descriptors are below:

```
Student_emasculate          PB_W5_noDEG_ZIP       10    0    3    1
```

`Student_emasculate` functional transformation name
`PB_W5_noDEG_ZIP` RMSD database (mean values, standard deviation and occurrence number for each 'word' in learning sample (e.g. 'AGGPL')
`10` Protein Block serial number ( PB 'i' in this case)
`0    3` considered interval in the chain (positions from the current to +3)
`1` the power of descriptor (linear, quadratic, square root, etc)

```
Log_occurence_differrence   PB_W5_noDEG_ZIP_denom  7    0.5
```

`Log_occurence_differrence` functional transformation name
`PB_W5_noDEG_ZIP_denom` RMSD database (mean values, standard deviation and occurrence number for each 'word' in learning sample (e.g. 'AGGPL')
`7` Protein Block serial number ( PB 'g' in this case)
`0.5` the power of descriptor (linear, quadratic, square root, etc)

```
T_statistics_window         PB_W7_trivial_PG  3    -2    2    2.7
```

`T_statistics_window` functional transformation name
`PB_W7_trivial_PG` RMSD database (mean values, standard deviation and occurrence number for each 'word' in learning sample (e.g. 'AGGPL')
`3` Protein Block serial number ( PB 'd' in this case)
`-2    2` considered interval in the chain (positions from –2 to +2)
`2.7` the power of descriptor (linear, quadratic, square root, etc)

```
T_statistics_single         PB_w9_tail  11    1.3
```

`T_statistics_single` functional transformation name
`PB_w9_tail` RMSD database (mean values, standard deviation and occurrence number for each 'word' in learning sample (e.g. 'AGGPL')
`11` Protein Block serial number ( PB 'h' in this case)
`1.3` the power of descriptor (linear, quadratic, square root, etc)

The Python implementation of all functional transformations can be found in the files located at

```
BasicDescriptors/Three_Letter_Mode/<functional_transformation_name>.py
```
