# Supplementary material for: TruMPET: A New Method for Protein Secondary Structure Prediction Using Neural Networks Trained on Multiple Pre-Selected Physicochemical and Structural Features
Source: Int J Mol Sci. 2025 Nov 21;26(23):11284. doi: 10.3390/ijms262311284 (PMC12692721; doi:10.3390/ijms262311284)
Supplement: Supplementary file 1 [file ijms-26-11284-s001.zip › Supplement S5.Descriptors.Preselection.pdf]

1. Databases preparation

Before running any scripts, carefully check all directory paths and file names.
You will need **frequency extrapolation databases** and the **AAindex** database for descriptor generation, neural network training, and protein structure prediction.
Precomputed databases (≈7.5 GB) are available for download at:
https://ftp.eimb.ru/Milch/TruMPET.2025/Databases/TruMPET2025.databases.tar.xz
Unpack this archive into the `Databases` directory. Alternatively, databases can be recomputed directly from the PDB databank following either the short or detailed manual:

- **Short guide:** https://ftp.eimb.ru/Milch/Generate.DB.Predictors/readme.txt
- **Full manual:** https://ftp.eimb.ru/Milch/Generate.DB.Predictors/Generate.Databases.and.Predictors.pdf

If only frequency databases are required, you may stop after **Step 2: `MakeFrequencyDatabases`**.
In addition to the databases, a list of protein chains filtered by X-ray resolution and sequence homology is needed. This list can be generated using the PISCES server:
https://dunbrack.fccc.edu/pisces/PISCES_OptionPage.php
We retrieved a non-redundant set of protein chains from the PISCES server [89] using the following filtering criteria: sequence identity ≤ 40%, resolution ≤ 3.0 Å, sequence length between 40 and 10,000 residues, R-factor ≤ 0.3, and X-ray structures only. The dataset was generated on July 7, 2025, initially contained 26,622 protein chains and randomly divided to training (`train.txt`) and validation (`validation.txt`) chains lists files.

2. DSSP structures downloading

Eight-class DSSP annotations for the selected protein chains can be obtained from the PDB-REDO DSSP download server [91] or generated locally using **mkdssp 4.1.3–4.5.5** (https://github.com/PDB-REDO/dssp).
To download precomputed DSSP files, run

```
python3 01.download.DSSP.py train.txt
```

where `train.txt` is the training dataset chains list. By default all files will be stored in the `DSSP` subdirectory of the current directory.

3. Preparation of the AA3_AA1_SS Database

Convert downloaded DSSPs to AA3_AA1_SS database using:

```
python3 02.convert.DSSP.py train.txt
```

where `train.txt` is the training dataset chains list. Ensure all paths are correct. By default, CIF-DSSP files are read from `DSSP/`, and converted files are written to `AA3_AA1_SS/` subdirectories.

4. Descriptor taskfile Preparation

Populate a `taskfile` (e.g., `current_ADD.task)` with all potential descriptors, transformations, and parameters based on structural and physicochemical considerations. This can be automated using `python3 03.generate.task.py descriptors.template`.
where `descriptors.template` is the file, containing template with parameter limits. The physicochemical parameters utilized from AAindex database are stored in file `AAindex.lst,` path to this file can be changed in `descriptors.template`. Each template file may define only one descriptor type. If multiple descriptor types are required, prepare separate templates and merge their task files prior to SDA analysis.

5. Data Preparation for Stepwise Discriminant Analysis (SDA)

Convert protein secondary structures extracted from CIF files into NumPy datasets:

```
python3 04.protchains.py train.txt
```

or run the parallelized version:

```
./run_parallel.01.sh
```

This step reads the `current_ADD.task` file and produces multiple .npy files in the output directory. Finally, combine the generated .npy files into a single CSV:

```
python3 05.make.dacsv.py
```

to convert all .npy files from directory `output` to `da.csv` file

6. Stepwise Discriminant Analysis (SDA)

Run SDA using:

```
./06.DAS_release da.csv taskfile
```

The analysis yields a list of statistically significant, mutually uncorrelated descriptors in `da.single_calculation_var_significant` file. SDA parameters can be tuned in the file `dia_subtle.option`; for example, it is recommended to test FISHER_FOR_SINGLE_CALC values between 100 and 1000 with a step of 100.

7. LDA Task File Generation

Generate the LDA task file containing significant descriptors:

```
07.make.ldatask.py da.single_calculation_var_significant
```

8. Data Preparation for Linear Discriminant Analysis (LDA)

Create NumPy datasets for LDA:

```
python3 08.protchains.py train.txt
```

Output will be written to the `output.lda/` directory. Parallel execution is available via:

```
./run_parallel.02.sh
```

This step will populate `output.lda` directory with the collection of .npy files for LDA based upon SDA-calculated task file and initial `train.txt` file.

9. Conversion to LDA CSV Format

Combine .npy files from output.lda/ into a single CSV file:

```
python3 09.make.ldacsv.py
```

10. Linear Discriminant Analysis (LDA)

Run LDA:

```
python3 10.lda.py
```

This step will be computationally intensive. Two output files will be generated: `greedy_selected_features.txt` and `greedy_selection_history.csv`. The first file contains list of the most significant, uncorrelated descriptors ranked by impact. The second file provides the data for a chart showing the accuracy increase per descriptor, as well as the total accuracy of the ensemble composed of the first *N* descriptors.

11. Iterative Refinement and Model Merging

Steps 4–10 can be iteratively repeated, guided by hypotheses regarding factors influencing protein secondary structure formation. Descriptor sets from different iterations should be merged into a single lda.task file, removing exact duplicates. The merged set is then subjected to an additional SDA (Steps 4–6) to select the final set of statistically significant, uncorrelated descriptors and determine their ranking.
