# Supplementary material for: TruMPET: A New Method for Protein Secondary Structure Prediction Using Neural Networks Trained on Multiple Pre-Selected Physicochemical and Structural Features
Source: Int J Mol Sci. 2025 Nov 21;26(23):11284. doi: 10.3390/ijms262311284 (PMC12692721; doi:10.3390/ijms262311284)
Supplement: Supplementary file 1 [file ijms-26-11284-s001.zip › Supplement S7.Network.pdf]

Supplement S7. LDA-Based Model Conversion and Neural Network Training

1. Converting LDA-Substantiated Descriptors to NumPy Datasets

The list of LDA-substantiated descriptors (see Supplement S5) must first be converted into a collection of NumPy datasets. You should rename result of LDA (`greedy_selected_features.txt` file) to `postLDA.task` file, remove the first string with the columns description and run supporting script

```
./11.makeNPY.sh
```

You must provide the `allchains.txt` file, which represents the union of the training and validation chains lists (see Supplement S5).

2. Neural Network Training

Once NumPy datasets are prepared, run the neural network training script:

```
python3 12.LSTM.py
```

Tested environments: Python 3.8, 3.10, 3.12
pytorch 1.12.1, numpy 1.24.4, scipy 1.10.1, numba 0.58.1, fair-esm 2.0.0
pytorch 2.5.1, numpy 2.2.6, scipy 1.16.1, numba 0.61.2, fair-esm 2.0.0
Hyperparameters (e.g., number of layers, hidden unit size, dropout, weight decay, learning rate) can be directly adjusted in `12.LSTM.py`. GPU usage is strongly recommended.

3. Model Output

Upon completion, two model files will be generated:
`512_2_cpu.pt` — CPU-optimized model
`512_2_cuda.pt` — GPU-optimized model
Move both files, along with the `postLDA.task`, into the directory `Models/LDA/`
These models can then be used for prediction as described in Supplement S10 (“Usage”).
