# Supplementary material for: TruMPET: A New Method for Protein Secondary Structure Prediction Using Neural Networks Trained on Multiple Pre-Selected Physicochemical and Structural Features
Source: Int J Mol Sci. 2025 Nov 21;26(23):11284. doi: 10.3390/ijms262311284 (PMC12692721; doi:10.3390/ijms262311284)
Supplement: Supplementary file 1 [file ijms-26-11284-s001.zip › Supplement S9.ESM2.4layer.pdf]

Supplemental Table S9. Results of benchmarking the ESM2 models on various test datasets with 4-layered bidirectional LSTM network, hidden unit size=1024.

**Dataset**	Q8, 650M	$F_{1:8}$, 650M	Q3, 650M	$F_{1:3}$, 650M	Q8, 3B	$F_{1:8}$, 3B	Q3, 3B	$F_{1:3}$, 3B	Dataset size, chains
Validation	0.7948	0.6449	0.8841	0.8829	0.7592	0.5790	0.8736	0.8732	5325
CB513	0.8008	0.6778	0.8913	0.8906	0.7693	0.6035	0.8800	0.8792	435*
TS115	0.7798	0.6191	0.8704	0.8659	0.7523	0.5600	0.8573	0.8526	115
TEST2018	0.7734	0.6192	0.8701	0.8682	0.7360	0.5408	0.8550	0.8534	245
TEST2020-HQ	0.6994	0.4765	0.8004	0.7976	0.6664	0.4267	0.7876	0.7850	124
CASP13	0.7547	0.5655	0.8477	0.8472	0.7031	0.5062	0.8212	0.8196	40
CASP14	0.6630	0.4370	0.7915	0.7917	0.6328	0.4009	0.7699	0.7702	34
CASP15	0.6838	0.4832	0.8068	0.8105	0.6584	0.4560	0.7981	0.8015	39

*In the CB513 dataset, many protein chains are split into domains and are considered as separate targets. Our prediction method, however, predicts on complete chains; thus, the chain number is less than 513, although all CB513 segments are considered (see Section 4.2). 1280 ESM2 embeddings obtained from esm2_t33_650M_UR50D model (33 layers, 650 million parameters) and 2560 ESM2 embeddings obtained from esm2_t36_3B_UR50D (36 layers, 3 billion parameters) model. esm2_t33_650M_UR50D model denoted as '650M', esm2_t36_3B_UR50D model denoted as '3B'.

The following **Table 4** from the main text of the manuscript is given for the comparison purpose.

Table 4. Results of benchmarking the TruMPET method on various test datasets.

**Dataset**	Q8, LDA	$F_{1:8}$, LDA	Q3, LDA	$F_{1:3}$, LDA	Q8, mix	$F_{1:8}$, mix	Q3, mix	$F_{1:3}$, mix	Dataset size, chains
Validation	0.8311	0.6949	0.9015	0.8992	0.8446	0.7137	0.9085	0.9062	5325
CB513	0.8557	0.7461	0.9148	0.9128	0.8624	0.7599	0.9175	0.9157	435*
TS115	0.8548	0.7411	0.9153	0.9096	0.8647	0.7628	0.9175	0.9119	115
TEST2018	0.8541	0.7518	0.9149	0.9122	0.8624	0.7671	0.9178	0.9150	245
TEST2020-HQ	0.8253	0.6882	0.8877	0.8841	0.8378	0.7091	0.8949	0.8911	124
CASP13	0.7876	0.6456	0.8700	0.8683	0.7957	0.6619	0.8704	0.8684	40
CASP14	0.7461	0.5438	0.8475	0.8472	0.7646	0.5668	0.8522	0.8513	34
CASP15	0.7286	0.5600	0.8338	0.8366	0.7357	0.5674	0.8402	0.8426	39
