# Supplementary material for: TruMPET: A New Method for Protein Secondary Structure Prediction Using Neural Networks Trained on Multiple Pre-Selected Physicochemical and Structural Features
Source: Int J Mol Sci. 2025 Nov 21;26(23):11284. doi: 10.3390/ijms262311284 (PMC12692721; doi:10.3390/ijms262311284)
Supplement: Supplementary file 1 [file ijms-26-11284-s001.zip › Supplement S10.Usage.pdf]

Supplement S10. Protein Secondary Structure Prediction by TruMPET 2025.

1. Databases preparation

You will need **frequency extrapolation databases** and the **AAindex** database for protein structure prediction. Precomputed databases (≈7.5 GB) are available for download at:
https://ftp.eimb.ru/Milch/TruMPET.2025/Databases/TruMPET2025.databases.tar.xz
Unpack this archive into the `Databases` directory.

2. Default path configuration

Before running any scripts, carefully check all directory paths and file names in files `TruMPET_cpu.py` and `TruMPET_gpu.py`. The default paths are the following:

```
MODEL_PATH       = "Models/mix/1024_4_cpu.pt"
TASK_SET_FILE    = "Models/mix/1024_4.task"
OUT_DIR          = "results"
LOG_LEVEL        = "INFO"
PATH_TO_FREQUENCY_STORE    = "Databases/FrequencyExtrapolation/"
PATH_TO_AAINDEX_FILE       = "Databases/AAindex/aaindex.txt"
PATH_TO_AAINDEX_TRI_LETTER = "Databases/AAindex/aaindex_mutant3.txt"
```

3. Protein secondary structure prediction (PSSP)

PSSP can be performed on CPU either on GPU. TruMPET2025 recognizes files in two formats: FASTA file – this mode performs protein secondary structure prediction without consideration of non-canonic amino acids; the input for prediction with consideration of non-canonic amino acids DATA file that must follow this structure:

- Line 1: Protein chain in three-letter amino acid codes, separated by spaces.
- Line 2: The same protein chain in one-letter amino acid codes, without spaces.
- No headers or comments are allowed in the file.

Example:

```
ALA GLY SER THR TYR
AGSTY
```

Usage examples:

```
./TruMPET2025_cpu.py -d 12ASA.data
./TruMPET2025_cuda.py -f 5B68_A.fasta
./TruMPET2025_cpu.py -f *.fasta
./TruMPET2025_cuda.py -d *.data
```

The results of PSSP are stored in the directory specified by the `OUT_DIR` variable (e.g., in the `results` subdirectory in the example above).
